# Dietary Protein Intake Is a Determining Factor for Skeletal Muscle Mass in Japanese Older People with Type 2 Diabetes: A Cross-Sectional Study

**DOI:** 10.3390/nu17040731

**Published:** 2025-02-19

**Authors:** Shota Moyama, Yuji Yamazaki, Takuya Takahashi, Noboru Makabe, Yoshiyuki Hamamoto, Takeshi Kurose, Yuichiro Yamada, Hitoshi Kuwata, Yutaka Seino

**Affiliations:** 1 Yutaka Seino Distinguished Center for Diabetes Research, Kansai Electric Power Medical Research Institute, Osaka 5530003, Japan; shota0531@gmail.com (S.M.); n-makabe@umin.ac.jp (N.M.); hamamoto.yoshiyuki@b4.kepco.co.jp (Y.H.); yamada.yuuichiro@a3.kepco.co.jp (Y.Y.); hitoshi.kuwa@gmail.com (H.K.); yutaka.seino.hp@gmail.com (Y.S.); 2 Center for Metabolism and Clinical Nutrition, Kansai Electric Power Hospital, 2-1-7 Fukushima, Fukushima Ward, Osaka 5530003, Japan; yzfr6_tt@yahoo.co.jp; 3 Center for Diabetes, Endocrinology and Metabolism, Kansai Electric Power Hospital, 2-1-7 Fukushima, Fukushima Ward, Osaka 5530003, Japan; kurose@nakanoshima-clinic.jp; 4 Nakanoshima Clinic, 2-1-2 Fukushima, Fukushima Ward, Osaka 5530003, Japan

**Keywords:** sarcopenia, protein intake, older people, type 2 diabetes, nutritional intervention, dietary pattern

## Abstract

**Background/Objectives**: In this study, we investigated the free-living nutritional intake of older people with type 2 diabetes (T2D) and examined the relationship between nutritional intake and skeletal muscle mass. **Methods:** Subjects aged 65 years or older with T2D who visited the Kansai Electric Power Hospital between 2015 and 2017 and had not yet received nutritional guidance or intervention at our hospital comprised the study group. Nutritional intake (energy, protein, lipid, and carbohydrate intake) was calculated from a 3-day dietary diary by the participants, and the relationship between nutritional intake and the skeletal muscle index (SMI) was retrospectively investigated. **Results:** In total, 91 subjects were recruited (53 males and 38 females, aged 70.3 ± 5.5 years). The energy and protein intakes were 28.7 ± 6.1 kcal/kg/day and 1.2 ± 0.3 g/kg/day, respectively. A significant positive correlation was found between the SMI and energy and protein intake (*p* < 0.001). Multiple regression analysis with the SMI as the dependent variable and age, gender, protein intake, lipid intake, and carbohydrate intake as the independent variables revealed protein intake to be an independent determinant of the SMI. **Conclusions:** In older people with T2D, the energy and protein intakes are likely to be lower than the recommended levels. Appropriate interventions for protein intake and energy intake are recommended to prevent loss of muscle mass in Japanese older people with type 2 diabetes.

## 1. Introduction

The incidence rate of sarcopenia in older people with diabetes is approximately three times that of non-diabetic individuals, partly due to the anabolic resistance and chronic inflammation caused by hyperglycemia. In addition, it has been reported that malnutrition and sarcopenia result in extended hospital stays and increased mortality [1,2]. The world population is aging, and the proportion of elderly people is expected to double from 12% in 2015 to 22% in 2050 [3]. These factors suggest that healthcare professionals should vigorously address sarcopenia in older individuals, especially in those with type 2 diabetes (T2D) or other non-communicable diseases (NCDs). Several studies have suggested targets for energy and protein intake that are necessary to maintain adequate physical function and prevent or improve sarcopenia; in the case of sarcopenia, it is generally necessary to consume at least 1.0 to 1.2 g/kg/day of protein, but 1.2 to 1.5 g/kg/day is recommended for people with acute or chronic disease and about 2.0 g/kg/day for those with severe inflammation [4]. We previously conducted a randomized controlled trial which indicated that intensive protein intervention to achieve a protein intake of 1.3 g/kg/day is effective in improving muscle strength and physical function in people aged 75 years or older with chronic diseases [5]. In addition, as anabolic resistance progresses with age, the ability of older people to synthesize their muscles is reduced. Moreover, muscle synthesis occurs primarily during the postprandial period of about 2–3 h [6,7]. Thus, in addition to ensuring an adequate total daily protein intake, the distribution of protein intake over the three meals is important to maximize muscle synthesis per day [4,6,8]. A recent previous study showed that in hospitalized older people, the higher the protein intake, the greater the lean mass [9]. Also, approximately half of the older people residing in nursing facilities have sarcopenia, and insufficient nutritional intake, particularly insufficient protein intake, has been cited as a risk factor between sarcopenia and malnutrition [10]. On the other hand, some studies have reported no correlation between nutritional intake and muscle mass in free-living older people who are outpatients [11,12]. Increased protein intake may be beneficial in improving sarcopenia in older people, but there is some ambiguity, and these previous studies were also based on data from individuals without diabetes. It is crucial to accumulate evidence on the actual daily nutritional intake of older people with T2D, and its relationship to muscle mass in a real-world setting. The primary objective of this study was to clarify the actual nutritional intake of Japanese older people with T2D who were free-living and had not received nutrition education. In addition, the secondary objective was to examine the association between nutritional intake (energy, protein, fat, carbohydrate) and skeletal muscle mass in this population. Therefore, the research questions of this study were as follows: What is the actual nutritional intake (especially protein intake) in Japanese older people with T2D, and is nutritional intake associated with skeletal muscle mass? This study describes the free-living nutritional intake of older people with T2D and evaluates the relationship between their total energy, protein, fat, and carbohydrate intake and their skeletal muscle mass.

## 2. Materials and Methods

### 2.1. Study Design and Subjects

This retrospective cross-sectional study was conducted in the Diabetes and Endocrinology and Metabolism Center of Kansai Electric Power Hospital (Osaka, Japan) from 2015 to 2017. This study was approved by the Ethical Committee of Kansai Electric Power Hospital (approval code: approval code: 24-120) and conducted in accordance with the Declaration of Helsinki (Fortaleza, revised in 2013), the Ethical Guidelines for Life Sciences, and Medical Research Involving Human Subjects (Ministry of Education, Culture, Sports, Science and Technology, Ministry of Health, Labour and Welfare, and the Ministry of Economy, Trade and Industry Notification No. 1, 2021). In addition, this paper was written in accordance with the Strengthening the Reporting of Observational Studies in Epidemiology (STROBE) statement and the STROBE checklist [13].

Inclusion criteria were those aged 65 years or older with T2D, and the exclusion criteria were as follows: (a) those who had received nutritional guidance, (b) those whose energy intake calculated from food records was less than 1000 kcal/day or more than 4000 kcal/day, (c) those with severe renal dysfunction (estimated glomerular filtration rate; eGFR less than 30 mL/min/1.73 m^2^), (d) those with a pacemaker or implantable cardioverter defibrillator, (e) those taking steroids, (f) those who were not independent in ADL, (g) those who had difficulty keeping a food record, (h) those with digestive disorders accompanied by digestive and absorption disorders, (i) those with mental illness, (j) those with malignant tumors, and (k) those with chronic inflammation such as chronic obstructive pulmonary disease. As this was an exploratory study, no sample size test was performed, and the maximum number of subjects who met the inclusion and exclusion criteria during the study period was selected as the study subject.

### 2.2. Measurements

Height and body weight were measured using an automatic measuring device (Tanita, Japan). Body fat mass, fat-free mass, and skeletal muscle index (SMI) were measured in the supine position after 15 min of bed rest using an InBody S10 (InBody Co., Seoul, Republic of Korea). A 3-day self-recorded food dairy and nutrition calculation software (Healthy Maker Pro 501 (Mushroom Soft Co., Ltd., Okayama, Japan)) were used to calculate energy, protein, fat, and carbohydrate intake per day. In addition, energy, protein, and fat intake at breakfast, lunch, and dinner were separately assessed. Because the 3-day food diaries are based on self-reporting and are prone to under-reporting or inaccurate reporting, especially in older adults, we met with all subjects in person to directly verify any discrepancies in the reported information. All data were retrospectively extracted from electronic medical records. The 3-day food diaries are used as Nutritional assessments in the National Institute for Longevity Sciences–Longitudinal Study of Aging (NILS-LSA) in Japan, and their validity has been guaranteed [14].

### 2.3. Statistical Analysis

Continuous variables such as age and weight were expressed as the mean with standard deviation; differences between males and females were compared using *t*-tests. The association between the SMI and nutritional intake was evaluated using Pearson’s product–moment correlation coefficient. In addition, multiple regression analysis with stepwise methods was performed with the SMI as the dependent variable and age, gender, nonprotein calories, protein intake, lipid intake, and carbohydrate intake as independent variables to analyze the factors determining the SMI. In addition, one-way analysis of variance was used to compare protein intake for the three meals (breakfast, lunch, and dinner), and Tukey’s test was used for multiple comparisons. Those with missing data on survey items were excluded from the analysis. The significance level for all statistical analyses was set at 5% on both sides, and *p*-values less than 0.05 were considered statistically significant.

## 3. Results

**Figure 1:**
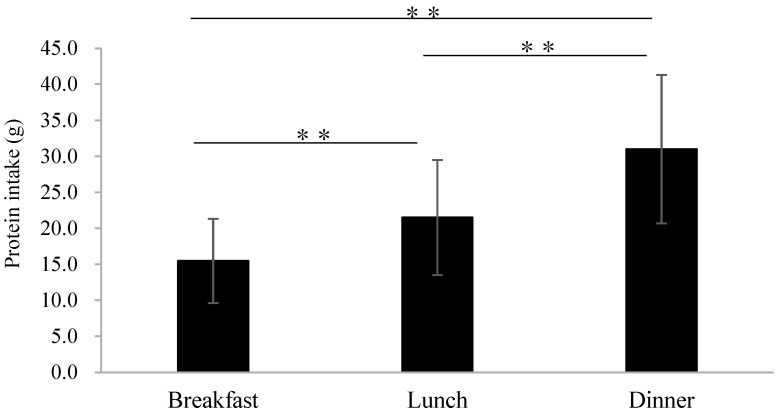
Comparison of protein intake for breakfast, lunch, and dinner. One-way analysis of variance (Tukey’s test was performed for multiple comparisons), ** *p* < 0.01.

In total, 91 subjects aged 65 years or older with T2D who had not yet received nutritional guidance or intervention at our institution were enrolled (53 males and 38 females, age 70.3 ± 5.5 years); their characteristics are shown in Table 1. There were no significant differences in age or BMI between males and females, but body fat mass was higher in females and lean mass was higher in males (*p* = 0.017, *p* < 0.001, respectively). In contrast, the proportion of subjects with an SMI below the diagnostic criteria for sarcopenia proposed by the Asian Working Group of Sarcopenia (SMI: <7.0 kg/m^2^ for males and <5.7 kg/m^2^ for females) was 30.2% for males and 44.7% for females; although the proportion of females with muscle mass loss tended to be higher, this difference was not statistically significant. Blood biochemistry tests showed that HbA1c also tended to be higher in females, while eGFR was similar, and there was no difference in renal function between the genders. While energy intake was significantly lower in females than in males, there were no significant differences between genders after making an adjustment for body weight. There were also no significant differences in protein or fat intake between the genders, but carbohydrate intake was higher in males (*p* = 0.013). Both genders showed energy intake below the target for adequate nutrition (30–35 kcal/kg/day) [4], while the majority of males and half of the females showed protein intake below the target for preventing or treating sarcopenia (1.2–1.5 g/kg/day) [4]. No significant difference was found between the genders regarding total energy intake, but females tended to have a higher percentage from snacks. Figure 1 shows a comparison of protein intake of the three meals (breakfast, lunch, and dinner). Protein intake varied significantly across the three meals, with breakfast having the lowest intake (16.0 ± 6.4 g in males and 14.7 ± 5.0 g in females). Additionally, there was no significant difference between males and females in the prevalence of diabetes-related complications such as neuropathy, retinopathy, and nephropathy, as well as other comorbidities. Regarding medications, the proportion of females taking antihyperlipidemic drugs was higher than males, but this difference was not statistically significant. Also, there were no differences between the genders in the proportion of other medications taken.

**Figure 2:**
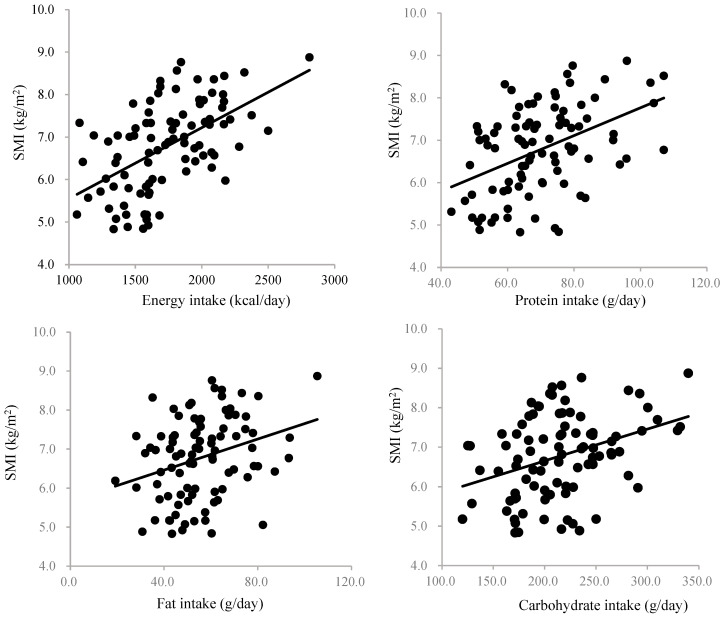
Relationship between nutrition intake and skeletal muscle index. SMI: skeletal muscle index.

Relationships between nutritional intake and the SMI are shown in Figure 2. The SMI showed significant positive correlations between energy intake (R = 0.522, *p* < 0.001), protein intake (R = 0.401, *p* < 0.001), fat intake (R = 0.300, *p* = 0.004), and carbohydrate intake (R = 0.358, *p* < 0.001). We conducted a multiple regression analysis with the SMI as the dependent variable and age, gender, nonprotein calories, protein intake, lipid intake, and carbohydrate intake as the explanatory variables. The results show that age and gender are independent determining factors of the SMI (Table 2). Protein intake was the only nutrition-related independent determinant of the SMI.

## 4. Discussion

### 4.1. Perspectives for Clinical Practice

This study retrospectively investigated the free-living nutritional intake of older Japanese people undergoing initial consultation for T2D who had not yet received nutritional dietary guidance and examined the relationship between nutritional intake and skeletal muscle mass. The data show that the majority of males and approximately half of the females fall below the recommended nutritional intake (energy 30–35 kcal/kg/day, protein 1.2–1.5 g/kg/day) [4], posing a risk for sarcopenia. Previous research has suggested that people with chronic diseases such as diabetes may need to consume more than 1.5 g/kg/day of protein [4], so the elderly population who are below the protein intake required to prevent sarcopenia is potentially higher than assumed. The present study demonstrates a statistically significant relationship between protein intake and the SMI independent of total energy intake, which strongly supports focusing on both protein intake and total energy intake during dietary intervention. Although several studies have suggested that the intervention to increase protein intake helps maintain and increase skeletal muscle mass or improve sarcopenia in older people, these participants are hospitalized or living in facilities, and these are people without diabetes [9,10]. A few reports have examined the relationship between protein intake and muscle mass or the presence or absence of sarcopenia in older people with diabetes who visit the hospital as outpatients [15,16,17]. However, there is little information on the relationship between daily food intake and muscle mass before those who start diet therapy under nutritional education. This is the first study to show the relationship between protein intake and skeletal muscle mass in Japanese older people with T2D in a real-world setting. The relationship between protein intake and skeletal muscle mass has been examined in middle aged people and non-diabetic older people, suggesting a relationship in which higher protein intake inhibits skeletal muscle loss, and our study is consistent with previous research [10,18,19]. Therefore, “protein” is a factor to consider in dietary interventions to prevent and improve sarcopenia in older people with T2D.

Both genders in this study showed similar dietary patterns, with lower protein intake at breakfast and higher intake at dinner. The skewed patterns of protein intakes in a day might lose a chance to make muscle due to an insufficient enhancement of muscle synthesis, which can affect the balance between muscle synthesis and muscle breakdown. In addition, older people experience a general decline in digestive and absorptive abilities and a decline in muscle protein synthesis abilities (anabolic resistance) as they age [7,8]. In the older population, the skewed pattern can have a striking impact. Therefore, it is thought to be important to not only ensure that the total amount of nutrition consumed each day is sufficient but to also equalize the amount of nutrition consumed in each of the three meals a day (breakfast, lunch, and dinner) [19,20,21]. However, because these previous studies [19,20,21] were conducted on non-diabetic individuals, it is unclear whether similar trends would be observed in diabetic individuals. The results of this study suggest that older people with T2D who have no history of nutritional guidance do not only not meet the daily energy and protein intake targets necessary for preventing and improving sarcopenia [4] but also have an uneven intake of nutrients over their three meals, which has implications for the forthcoming nutritional guidance for these patients with T2D.

### 4.2. Limitations

This study has several limitations. First, although the results of the multivariate analysis indicate that protein intake is an independent determining factor for the maintenance of and improvement in the SMI, this study is a cross-sectional study conducted at a single facility and therefore cannot establish a causal relationship. Verification of the findings by multi-center and intervention studies is required. Second, since physical activity was not assessed in this study, we could not adjust for exercise activity in the multivariate analysis. Finally, while the subjects in this study had not undergone their first T2D consultation at our hospital, they may have obtained any nutritional or dietary intervention before visiting our hospital, meaning that bias regarding nutritional intervention could not be completely eliminated.

## 5. Conclusions

In a group of people aged 65 years or older with T2D who had not received nutritional guidance, the percentage of participants meeting target energy and protein intake sufficient to prevent or improve sarcopenia was low. Furthermore, we found that adequate protein intake was associated with better skeletal muscle mass maintenance and improvement, highlighting the importance of sufficient protein consumption in this population.

## Figures and Tables

**Table 1:** Characteristics of study subjects.

	All Participants	Male	Female
Number of Participants	91	53	38
Age (year)	70.3 ± 5.5	70.4 ± 5.4	70.3 ± 5.6
Height (cm)	159.9 ± 9.1	165.2 ± 7.1	152.4 ± 5.5
Body weight (kg)	61.5 ± 11.4	64.8 ± 9.7	56.7 ± 12.1
BMI (kg/m^2^)	24.0 ± 3.6	23.7 ± 2.9	24.3 ± 4.4
Body fat mass (kg)	19.6 ± 7.3	18.0 ± 5.9	21.7 ± 8.4
Fat-free mass (kg)	41.9 ± 8.0	46.8 ± 6.0	35.0 ± 4.8
SMI (kg/m^2^)	6.8 ± 1.0	7.3 ± 0.8	6.0 ± 0.8
HbA1c (%)	7.6 ± 1.1	7.4 ± 1.0	7.9 ± 1.1
Cr (mg/dL)	0.84 ± 0.28	0.95 ± 0.95	0.69 ± 0.18
eGFR (mL/min/1.73 m^2^)	66.4 ± 17.3	65.3 ± 16.5	68.0 ± 18.4
Energy intake (kcal/day)	1726.0 ± 340.3	1828.3 ± 326.0	1583.2 ± 310.4
Energy intake (kcal/kg/day)	28.7 ± 6.1	28.4 ± 4.7	29.0 ± 7.7
Protein intake (g/day)	69.7 ± 14.4	72.1 ± 14.1	66.2 ± 14.2
Protein intake (g/kg/day)	1.16 ± 0.29	1.13 ± 0.24	1.21 ± 0.34
Fat intake (g/day)	55.9 ± 15.8	57.5 ± 15.8	53.6 ± 15.8
Carbohydrate intake (g/day)	215.6 ± 46.6	225.7 ± 48.6	201.4 ± 40.1
Ratio of energy intake in breakfast (%)	24.5 ± 7.3	24.5 ± 8.3	24.4 ± 5.6
Ratio of energy intake in lunch (%)	31.7 ± 7.3	31.1 ± 8.9	32.5 ± 4.2
Ratio of energy intake in dinner (%)	39.0 ± 8.6	40.3 ± 9.7	37.0 ± 6.4
Ratio of energy intake in snack (%)	4.9 ± 5.3	4.0 ± 4.9	6.1 ± 5.6
Comorbidity			
Diabetic neuropathy [n (%)]	57 (62.6)	32 (60.4)	25 (65.8)
Diabetic retinopathy [n (%)]	42 (46.2)	24 (45.3)	18 (47.4)
Diabetic nephropathy [n (%)]	29 (31.9)	17 (32.1)	12 (31.6)
Hypertension [n (%)]	56 (61.5)	33 (62.3)	23 (60.5)
Dyslipidemia [n (%)]	52 (57.1)	30 (56.6)	22 (57.9)
Chronic kidney disease [n (%)]	13 (14.3)	8 (15.1)	5 (13.2)
Heart failure [n (%)]	4 (4.4)	2 (3.8)	2 (5.3)
Cardiovascular disease [n (%)]	19 (20.9)	10 (18.9)	9 (23.7)
Malignant tumors [n (%)]	0 (0.0)	0 (0.0)	0 (0.0)
Medications			
Antihypertensive drugs [n (%)]	48 (52.7)	27 (50.9)	21 (55.3)
Antihyperlipidemic drugs [n (%)]	46 (50.5)	24 (45.3)	22 (57.9)
Antihyperglycemic drugs [n (%)]	77 (84.6)	44 (83.0)	33 (86.8)
Insulin [n (%)]	27 (29.7)	17 (32.1)	10 (26.3)
Steroid [n (%)]	0 (0.0)	0 (0.0)	0 (0.0)

Data are presented as the mean ± standard deviation. BMI: body mass index; SMI: skeletal muscle index; HbA1c: hemoglobin A1c; Cr: creatinine; eGFR: estimated glomerular filtration rate.

**Table 2:** Multiple regression analysis of influence factors on skeletal muscle index.

	Model 1		Model 2	
	Standardized Regression Coefficient	*p*-Value	Standardized Regression Coefficient	*p*-Value
Age	-	-	−0.265	0.001
Gender	−0.640	<0.001	−0.602	<0.001
Nonprotein calories	-	-	-	-
Protein intake	-	-	0.193	0.015
Fat intake	-	-	-	-
Carbohydrate intake	-	-	-	-

Gender was defined as male (1) or female (2).

## Data Availability

The datasets generated during and/or analyzed during the current study are available from the corresponding author on reasonable request.
